# Risk of significant cytopenias after treatment with tocilizumab in systemic juvenile arthritis patients with a history of macrophage activation syndrome

**DOI:** 10.1186/1546-0096-10-30

**Published:** 2012-08-29

**Authors:** Elizabeth A Kessler, Sheetal S Vora, James W Verbsky

**Affiliations:** 1 Division of Rheumatology, Department of Pediatrics, Medical College of Wisconsin, Milwaukee, WI, USA; 2 Children’s Corporate Center, Suite C465 9000 West Wisconsin Avenue, Milwaukee, WI 53226, USA

**Keywords:** Juvenile idiopathic arthritis, Macrophage activation syndrome, Tocilizumab

## Abstract

Tocilizumab (TCZ) is the first FDA- approved treatment for systemic juvenile idiopathic arthritis (sJIA). We report 3 cases of cytopenias in children with sJIA treated with TCZ. Two of the children who developed significant cytopenias shortly after initiation of TCZ had a history of macrophage activation syndrome. We raise the possibility that patients with a tendency towards MAS have an increased risk of developing cytopenias when treated with tocilizumab.

## Background

Systemic juvenile idiopathic arthritis (sJIA) is a subtype of chronic childhood arthritis of unknown etiology that is characterized by spiking fever, rash, generalized lymphadenopathy, hepatosplenomegaly, and serositis [1]. About 7% of sJIA patients develop macrophage activation syndrome (MAS), which is due to excessive activation of macrophages and T cells leading to a profound inflammatory reaction. Like sJIA, MAS manifests as fever (though more persistent as compared to sJIA), lymphadenopathy, and hepatosplenomegaly. Additionally, patients with MAS can have significant cytopenias, liver dysfunction, and serious coagulopathies [2]. Tocilizumab (TCZ), an anti-interleukin (IL)-6 receptor antibody, is the first FDA- approved treatment for sJIA. Prior studies of TCZ have not reported the development of significant cytopenias requiring medication discontinuance [3-5]. We report 3 cases of cytopenias in children with sJIA treated with TCZ necessitating a dose reduction or discontinuation of the drug. The 2 children who had a history of recurrent MAS developed significant cytopenias soon after initiation of TCZ. All three children were dosed according to manufacture guidelines; for patients <30 kg, 12 mg/kg was given every two weeks and for those ≥ 30 kg, 8 mg/kg was given every two weeks.

## Case presentations

### Case 1

**Figure 1:**
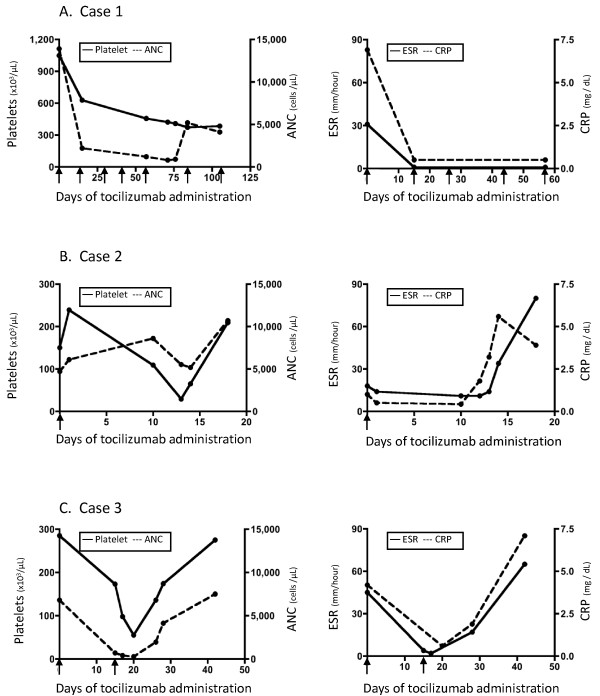
**Laboratory value responses to tocilizumab in Case 1 (A), Case 2 (B), and Case 3 (C).** Platelet counts and Absolute neutrophil counts (ANC) (left column), and erythrocyte sedimentation rate (ESR) and C-reactive protein (CRP) levels (right column) from initiation of tocilizumab at Day 0. Arrow indicates dosing of tocilizumab. Note differing x-axis intervals among the three cases.

A 6 year-old Caucasian girl with a 6 month history of sJIA was started on twice monthly TCZ therapy (12 mg/kg/dose) after failed treatment with methotrexate, adalimumab, and rilonacept due to persistent arthritis and inability to wean steroids. After 5 transfusions of tocilizumab over a twelve week period, her absolute neutrophil count (ANC) dropped from 13,900 to 800 cells/μL. Platelet count, erythrocyte sedimentation rate (ESR) and C-reactive protein concentration (CRP) normalized (Figure 1A). As the patient was otherwise clinically well when she became neutropenic, further laboratory studies were not obtained. Methotrexate and TCZ were held for two weeks until the neutropenia resolved. Methotrexate was resumed and her TCZ dose was subsequently decreased to 8 mg/kg/dose and administered every three weeks without further incident over a 10 month follow-up period.

### Case 2

A 3 year-old Caucasian girl had a 2 year history of sJIA, complicated by two previous episodes of macrophage activation syndrome (MAS) as well as chronic hepatitis with liver biopsy showing nonspecific chronic portal triaditis and minimal periportal reticulin fibrosis. Because she continued to exhibit fever, rash and serositis while on steroids and anakinra (up to 8 mg/kg/day), twice monthly TCZ was initiated at 12 mg/kg/dose and anakinra was discontinued. Two weeks after her initial infusion, the platelet count dropped from 150 k to 29 k/μL, while the ANC remained normal (Figure 1B). She had stable hepatosplenomegaly and her aspartate transaminase (AST) and alanine transaminase (ALT) were about twice normal, which was her baseline due to her chronic hepatitis. Infectious work-up for bacterial and viral sources, including adenovirus, Epstein-Barr virus (EBV), human herpesvirus 6 (HHV-6), cytomegalovirus (CMV), and enterovirus was negative. Platelets were administered, TCZ was held, and her prednisone dose (2 mg/kg) remained unchanged. Given the rapid decline in platelet count, we considered MAS. Her soluble interleukin-2 receptor (sIL-2R) level was 5451 U/ml (reference range 406–1100 U/ml). Although the hemoglobin dropped from 12.8 to 10.4 g/dL, fibrinogen went from normal to 173 mg/dL, and ferritin peaked at 12,900 ng/mL, these laboratory parameters improved without any therapeutic intervention. The patient’s thrombocytopenia eventually resolved and she subsequently developed labs indicative of a flare of sJIA: leukocytosis (white blood cell count increased from 8,500 to 17,100 cells/ μL), resolution of thrombocytopenia (platelet count went from 65 k after transfusion to 209 k/μL), and an increase in inflammatory markers (ESR went from 31 to 80 and CRP from 1.8 to 5.6 mg/dL). Due to the significant thrombocytopenia, TCZ was not restarted, but rather, anakinra was resumed.

### Case 3

A 15 year-old Caucasian male with a history of sJIA for 11 years had failed to achieve long-term remission with adalimumab, etanercept, cyclosporine, abatacept, anakinra, and IVIG. He’d had 3 previous episodes of MAS. After two doses of twice monthly TCZ (8 mg/kg/dose), his ANC dropped to 280 cells/μL, platelet count to 55 k/μL, and Hgb to 8.7 g/dL (Figure 1C). He had stable splenomegaly and his liver enzymes, CRP, ESR, ferritin, and fibrinogen were normal. Bacterial and viral studies, including EBV, HHV-6, CMV, enterovirus, and parvovirus B19 were negative. TCZ and cyclosporine (4 mg/kg/day) were held. Two weeks later the pancytopenia resolved and cyclosporine was resumed.

## Discussion

Three published studies have evaluated the efficacy and safety of TCZ in patients with sJIA [3-5]. A fourth study, the TENDER trial, is currently in the extension phase [6]. Patients with a history of MAS were included in two of the published studies as long as features of MAS were not present in the weeks leading up to initiation of TCZ [3,5]. The TENDER trial had no such exclusion criteria. Woo, *et al.* found that most children had lymphopenia after a single infusion of 2, 4, or 8 mg/kg of TCZ, although many had low lymphocyte counts prior to treatment. No other cytopenias were reported with the exception of transient pancytopenia in one patient at week seven, not requiring withdrawal from the study [4]. Yokota, *et al.* also used a dose of 2, 4, or 8 mg/kg of TCZ every two weeks for three total doses without report of cytopenias [5]. The most recently published study used 8 mg/kg of TCZ every two weeks with an open-label extension phase of at least 48 weeks. They had one patient who developed transaminitis and neutropenia (963 cells/μL) in association with EBV mononucleosis two weeks after the fifth infusion of TCZ. Laboratory values normalized after three weeks and the patient resumed treatment with TCZ [3].

Our experience in using TCZ has not been reflective of the published studies. We describe adverse reactions with significant cytopenias in three children treated with TCZ. Notably, two children (cases 2 and 3) who developed cytopenias with TCZ had a history of MAS. The cytopenias in cases 2 and 3 occurred after one to two doses, and in both instances, were characterized by thrombocytopenia. This is in contrast to case 1, who developed isolated neutropenia after her 5^th^ infusion. Case 2, in addition to thrombocytopenia, also developed anemia, hypofibrinogenemia, and hyperferritinemia, which could be consistent with MAS, although these lab abnormalities resolved without any intervention. Following this normalization, her laboratory values subsequently became consistent with an inflammatory response, suggesting that after a washout period of TCZ, her sJIA flared.

IL-6 is an important acute phase response protein that increases neutrophil and platelet counts, fibrinogen, and the ESR. The literature thus far has not described cytopenias requiring TCZ modification. However, our experience suggests that cytopenias with TCZ are more common than previously documented. One explanation for the discrepancy between the literature and our experience could be that some of the dosages used in these studies were lower than that currently recommended by the manufacturer. In the cases described above, treatment with TCZ resulted in cytopenias that were more rapid in onset in those with a prior history of MAS (cases 2 and 3). Notably, thrombocytopenia occurred with both of these children, which is in contrast to the isolated neutropenia seen in case 1. It is possible that cases 2 and 3 had subclinical MAS, which was balanced by the acute phase response. Addition of IL-6 blockade by TCZ altered this balance, leading to cytopenias. The concept of subclinical MAS is supported by the finding that children with sJIA who otherwise do not meet criteria for MAS demonstrate the presence of hemophagocytosis on bone marrow aspirate [7]. We do not believe TCZ induces MAS in such patients. Rather, the effects of TCZ can make it difficult to distinguish from ongoing hemophagocytosis in MAS. A recent publication has also suggested that TCZ can make MAS more difficult to diagnose due to alteration of laboratory studies [8]. As neutropenia can be seen after TCZ administration, perhaps development of thrombocytopenia is a more helpful marker of ongoing subclinical MAS as seen in our case series.

## Conclusions

Our experience suggests that additional caution in TCZ use for children with a history of MAS is warranted to avoid serious adverse events related to cytopenias. Such measures might include a dose reduction or modification of the frequency with which TCZ is given.

## Consent

A waiver of informed consent and waiver of Health Insurance Portability and Accountability Act authorization have been obtained through the local Institutional Review Board.

## Competing interests

The authors declare that they have no competing interests.

## Authors’ contributions

EK acquired the data, interpreted data and drafted the manuscript. SV and JV were involved in the interpretation of data and helped to draft the manuscript. All authors read and approved the final manuscript.
